# Efficacy of health literacy interventions aimed to improve health gains of higher education students—a systematic review

**DOI:** 10.1186/s12889-024-18358-4

**Published:** 2024-03-22

**Authors:** Jorge Rosário, Beatriz Raposo, Eunice Santos, Sónia Dias, Ana Rita Pedro

**Affiliations:** 1 Comprehensive Health Research Centre, University of Évora, Évora, Portugal; 2 Institute for Advanced Studies and Research, University of Évora, Évora, Portugal; 3 Polytechnic Institute of Beja, Beja, Portugal; 4 NOVA National School of Public Health, Public Health Research Centre, CISP, NOVA University Lisbon, Lisbon, Portugal; 5 NOVA National School of Public Health, Public Health Research Centre, Comprehensive Health Research Center, CHRC, NOVA University Lisbon, Lisbon, Portugal

**Keywords:** Health literacy, Heath literacy interventions, Health gains, Higher education students

## Abstract

**Background:**

Health literacy (HL) among higher education students is low, making them vulnerable about their health. To reverse this trend, higher education institutions promote HL interventions with various topics and methods. A comprehensive understanding of HL interventions is essential to determine whether these interventions meet the health information needs to improve health outcomes (health gains). The aim of this review was to identify and synthesise evidence on the efficacy of HL interventions implemented in academic settings to improve health outcomes.

**Methods:**

A systematic review was performed followed the PRISMA guidelines, protocol was registered in PROSPERO (CRD42022369869). A search strategy was performed in the EBSCO Host Web platform, the time limit placed was: 01/01/2017 to 30/09/2022. Eligible studies were those published in peer-reviewed journals and involved higher education students over the age of 18 as the subject of the intervention. Eligible interventions included any interventions evaluated in a study with comparison group that included a pre-post measure of health outcomes, were conducted in an academic setting. To methodology quality of included studies, it was used the Joanna Briggs Institute critical appraisal tool. To synthesise results narrative and thematic synthesis was conducted.

**Results:**

A total of 9 articles were included in this review, identified health literacy interventions with an impact on health outcomes. The total studies involved 2902 higher education students. All 9 studies were randomised controlled trials. The synthesised evidence supports the efficacy of interventions that contributed to positive changes in mental health, attitudes, norms, and self-efficacy of condom use, emotional, social, and psychological well being, subjective sleep quality, sleep latency, and habitual sleep efficiency, physical activity, and self-reported servings fried foods. HL interventions were educational or motivational and related to health promotion, disease prevention or healthcare.

**Conclusions:**

HL interventions in higher education students can significantly improve health outcomes protecting them from the negative effects of threats for their health. The interventions designed with different strategies are more effective. HL interventions are associated with health benefits on health promotion, disease prevention and healthcare. For the attendance of higher education to be a successful experience, continuity of HL interventions developed in academic settings is necessary.

## Background

Since 1970 a concept of health literacy (Simonds, 1974) has been the focus of attention in the field of public health and healthcare [1]. Health literacy is considered by the United Nations a valuable tool to improve communities’ health status and achieve sustainable development [2], has been recognised by the World Health Organization as essential to achieving the Sustainable Development Goals [3] and encompasses the personal characteristics and social resources of individuals and communities [4].

The European Health Literacy Project (HLS-EU) includes both a public health perspective on health literacy and an individual approach [1]. According to Sørensen et al. [1], it appears to be related to literacy and includes the "knowledge, motivation and skills to access, understand, evaluate and apply health information" to make judgements and decisions in everyday life about: (i) health care; (ii) disease prevention; and (iii) health promotion to "maintain or improve quality of life throughout life".

Nutbeam identified three dimensions of health literacy: functional (the ability to read health information and sometimes numeracy), interactive (literacy or cognitive skills), and critical literacy (a more advanced set of skills that, together with social skills, can be used to critically analyse and use information to gain greater control over life events and other situations) [5].

In interpreting the concepts of health literacy, we can see that it is a complex phenomenon that involves individuals, families, communities, and systems [6]. Health literacy depends not only on individual competence, but also on the environment, resources and context in which people live [7]. Organisational structures and the availability of resources influence health literacy (at the level of knowledge and skills) and people's ability to make health decisions (for their own health and wellbeing and that of those around them) [8].

Research has demonstrated that health literacy is a modifiable determinant of health and healthcare service utilisation [9, 10]. Health literacy is crucial for preventing noncommunicable diseases [11] and is independently linked to poorer chronic disease management and less effective drug use [12]. People with a low level of health literacy are less responsive to traditional health education methods and they make less use of preventive services, such as immunisation or screening [13].

According to research, higher education students exhibit lower levels of health literacy compared to other students of the same age [14]. For instance, a study conducted on Portuguese students found that 44% of them had problematic or inadequate health literacy levels [15]. University is a crucial transitional period for many students, as they move from adolescence to young adulthood and become more independent in making health-related decisions [16]. This period is also important for developing health literacy [17]. Students in higher education are receptive to information, making it more likely that healthy behaviours established during this phase of life will continue [18].

Health literacy is one of the few social determinants of health influenced by the individual or behavioural interventions to increase personal capabilities and be mitigated by reducing the situational demands experienced by people in different settings [8, 9]. The evidence suggests carrying out interventions that increase the health literacy of higher education students [19]. However, there are no systematic reviews that responded to this gap. Thus, herein, we conducted a systematic literature review to include studies that developed interventions to promote health literacy among higher students and measured health gains (effectiveness of health literacy interventions), with attention to rigour, clarity, and quality of the process. The aim of this systematic review was to identify and synthesise higher education students’ health gains, attributable to health literacy interventions in the academic setting. Given the understudied and specific scope of this study, the research question was formulated as a starting point: “What health gains are attributable to health literacy interventions implemented in an academic setting among university students?”.

This systematic review provides evidence on the nature and effectiveness of health literacy interventions implemented in academic settings that may be useful for planning future interventions. Knowing the characteristics of the interventions and the health outcomes they achieve will help us design interventions that impact health outcomes. Interventions to improve the health literacy in higher education students help empower them to make good health decisions.

## Materials and methods

This systematic review was conducted in accordance with the Declaration of *Protocols Preferred Reporting Items for Systematic Reviews and Meta-Analyses* (PRISMA) [20], the methodological design of the Joanna Briggs Institute (JBI) [21] and the PICOD model (Patient/Problem, Interventions, Comparison, Outcome, Design). It was registered in the International Prospective Register of Systematic Reviews (PROSPERO) under registration number CRD42022369869.

### Eligibility criteria

Eligible studies were those published in peer-reviewed journals and involved higher education students over the age of 18 as the subject of the intervention.

Following the PICOD principles, the inclusion criteria were: (1) Patient/Problem: studies including higher education students, from the public or private sector, targeting health literacy interventions; (2) Interventions: health literacy interventions carried out in an academic setting, motivational, behavioural, psychoeducational or others; (3) Context: Health literacy interventions in an academic setting, health outcomes measured in the intervention and control groups, and pre-post intervention outcomes; (4) Outcomes: Health outcomes (primary and secondary) and health gains as outcomes in health promotion, disease prevention, use of health services; (5) Study design: qualitative, quantitative, and mixed primary studies, randomised controlled trials, quasi-experimental, experimental studies, comparison group studies. Studies published in English, French, Spanish and Portuguese were also included.

Exclusion criteria were: (1) studies with students outside higher education, public or private sector; (2) higher education students' health gains resulting from health literacy interventions that have not been carried out in an academic environment; (3) interventions delivered during course attendance, not in an academic setting; (4) secondary studies, integrative literature reviews, scoping reviews, systematic literature reviews.

### Search strategy

A search was carried out on the EBSCO Host Web platform in the databases: *Academic Search Complete, Business Source Complete, CINAHL Plus with Full Text, ERIC, Library, Information Science & Technology Abstracts, MedicLatina, MEDLINE with Full Text, Psychology and Behavioral Sciences Collection, Regional Business News, SPORTDiscus with Full Text, Teacher Reference Center,* with the following descriptors in the “Medical Subjects Heading (MeSH”) and with Boolean operators (AND or NOT): Higher Education(= University), Universities, Students, Health Literacy, Health Education, Health Knowledge, Health Promotion, Health Programs, Health Services, Randomised Controlled Trial and Health. The following combination of terms was performed: [(Higher education OR Universities OR students) AND (Health literacy OR Health Education OR Health Knowledge OR Health) AND (Health Promotion OR Health programs OR Health services OR Program) AND (RCT OR Randomised Control Trials OR Randomised Controlled Trials)]) AND TI randomised AND AB Universities NOT TI Systematic Review. As limiters, we defined having full text, the timeline 01/01/2017 to 30/09/2022, being students aged 18 or over, and having as a linguistic limitation the inclusion of articles in English, French, Spanish and Portuguese. Manual searches were conducted in grey literature databases, including Bielefeld Academic Search Engine, GreySource, and CORE, as well as in dissertation databases. The primary search was conducted independently by two researchers (J.R., B.R.).

### Selection process

The study selection was made in two phases. The first phase consisted of the selection of the title and abstract, and the second phase was the selection of the remaining articles based on the full text. The selection process was independently applied by two researchers (J.R., B.R.), manually, without resorting to any software or program. The abstracts were systematically screened based on in- and exclusion criteria (J.R., A.R.P.). When a study did not meet the inclusion criteria, it was excluded, and the following study was screened. In articles whose titles and/or abstracts raised doubts, the full text was read. The researchers (J.R., B.R.) compared their selections blindly, with later clarification of the differences using a third researcher (A.R.P.).

From all the selected articles, the full text was obtained, and after reading it, the list of compliance with the inclusion and exclusion criteria, the adequacy of the keywords, and answer to the research question were applied. All studies were evaluated with the Template for Intervention Description and Replication—TIDieR [22].

### Methodological quality

The methodological quality of the selected studies was evaluated, in order to minimise bias, using the Joanna Briggs Institute critical appraisal tool [23].

Two investigators independently assessed the quality of the articles (J.R., B.R.). Differences that arose in the assessment of the methodological quality of the studies were clarified by a third investigator (A.R.P.).

### Data extraction and synthesis

The data were extracted using a table to systematically identify the characteristics of the studies. The following data were extracted: (i) authors, year; (ii) country of study; (iii) title, participants; (iv) objective; (v) methodology; (vi) Interventions; (vii) description of the main results/conclusions.

A thematic synthesis was carried out in the review of the articles (J.R.,B.R.,E.S.,S.D.,A.R.P.). In the full reading of the articles, relevant data were highlighted. After a second reading, we tried to identify the answer to the research question “line by line”, coding each answer according to the “theme/idea/area” of the data obtained. The synthesis process consisted of transposing the codes between the studies. After the health gains were coded, the correspondence between the selected text and the codes was checked. Codes were generated that were grouped to create themes/meaning units after descriptive themes were obtained. In the final phase, "generic" themes were created with sub themes that included descriptive themes and went beyond the primary studies to answer the research question topic (some themes are generic because they are broad themes).

## Results

**Fig. 1:**
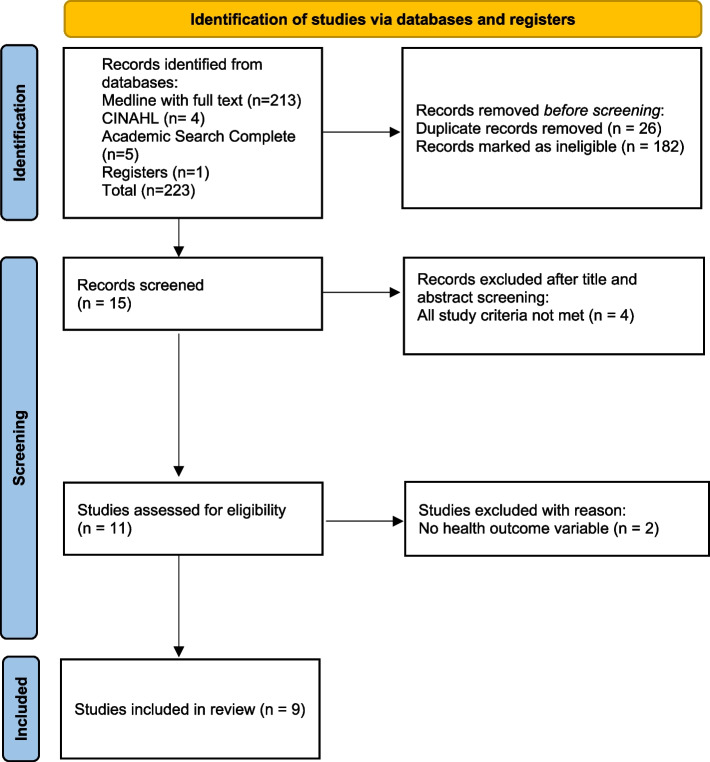
PRISMA flow diagram

Of the 223 results obtained by reading the abstract and title and subsequent application of the inclusion and exclusion criteria, 182 articles were eliminated, and all the remaining articles identified were retrieved. Of the 41 articles, were excluded: 26 duplicates, 4 did not contain all the study criteria, and 2 did not have outcome variables of health gains. After this screening and analysis, 9 articles [24–32] were selected that will constitute the body of analysis of the present study according to the PRISMA diagram (Fig. 1).

All 9 studies were randomised controlled studies, one published in 2017 [29], three in 2018 [25, 30, 31], one in 2019 [32], one in 2020 [24] and three in 2021 [26–28]. All studies were published in English. They were carried out in the following countries or regions: Germany [28], Australia [32], France [27], Hong Kong [26], Israel [31], United States of America [30], Macau [25], Pennsylvania [29], and Sweden [24]. Three studies were distributed among the European [24, 27, 28], three among the Asian [25, 26, 31], one among the Oceanic [32], and two among the American [29, 30] continents.

The studies involved 2902 among higher education students, distributed 2693 in university education and 209 in colleges. The smallest sample size is 22 students, and the largest is 746. The mean age of the students was 24 years. The number of participants in the intervention groups is 1641 students, and in the control groups it is 1261.

In the analysis of the studies, we sought to know the health gains of higher education students after participating in health literacy interventions carried out in an academic environment. The possibility of a meta-analysis was considered, but given the heterogeneity of the interventions, differences in the follow-up time, and the way of measuring the results, we decided to abandon this option. For data synthesis, we carried out a narrative synthesis (narrative analysis/narrative synthesis). Thematic synthesis was also done.

Studies were grouped by the research theme, and the following were identified: (i) mental health [24, 25, 27, 32]; (ii) sexual health literacy [26]; (iii) use of electronic mental health services [28]; (iv) physical activity [29, 30]; (v) reduction of anxiety in tests and related/associated symptoms [31], and (vi) sleep quality [25]. The analysed studies showed homogeneity in terms of objectives, most of which evaluated the effectiveness of interventions carried out in an academic environment on health outcomes. Regarding data collection methods, the authors applied electronic questionnaires to assess socio-demographic variables and outcomes through measurement scales.

When analysing the methodological quality of the studies, we followed the cohort points defined by Coughlan and Cronin which were 6 and 10 [33]. The authors defined three levels of quality. Each quality level was thus defined according to compliance with the evaluation criteria: low quality (compliance with 0 to 5 criteria), medium quality (compliance with 6 to 10 criteria), and high quality (compliance with 11 to 13 criteria). All studies met more than 70% of the criteria (JBI): one study with 76,9% [25], one study with 84,6% [31], 4 studies with 92,3% [24, 26–28], and 3 studies with 100% [29, 30, 32]. All studies have methodological quality, and by applying the scale of Coughlan and Cronin, 8 [24, 26–32] out of 9 studies were of high quality. It should be noted that three studies [29, 30, 32] meet all methodological quality assessment criteria, four studies [24, 26–28] meet 92.3% of the criteria, one meets 84.61% [31], and one meets 76.92% [25]. The evaluation of the methodological quality of the studies was carried out through the application of the Joanna Briggs Institute appraisal criteria. The Table 1 shows the summary of the evaluation of the methodological quality of the studies (JBI level of evidence for RCT).

**Table 1:** Assessed using JBI critical appraisal checklist for randomized controlled trials

Study	Randomization	Allocation	Baseline characteristics	Treatment pre/post	Participant blinding	Delivery blinding	Assessor blinding	Follow up complete	Group analysis	Outcome measurement	Reliable measurement	Statistical analysis	Appropriate trial design	%	Y-yes	N–no	U-unclear
[31]	Y	Y	Y	U	U	Y	Y	Y	Y	Y	Y	Y	Y	84,6	11	0	2
[28]	Y	Y	Y	Y	Y	U	Y	Y	Y	Y	Y	Y	Y	92,3	12	0	1
[24]	Y	Y	Y	Y	Y	Y	U	Y	Y	Y	Y	Y	Y	92,3	12	0	1
[25]	Y	Y	Y	U	U	U	Y	Y	Y	Y	Y	Y	Y	76,9	10	0	3
[29]	Y	Y	Y	Y	Y	Y	Y	Y	Y	Y	Y	Y	Y	100	13	0	0
[30]	Y	Y	Y	Y	Y	Y	Y	Y	Y	Y	Y	Y	Y	100	13	0	0
[27]	Y	Y	Y	U	Y	Y	Y	Y	Y	Y	Y	Y	Y	92,3	12	0	1
[32]	Y	Y	Y	Y	Y	Y	Y	Y	Y	Y	Y	Y	Y	100	13	0	0
[26]	Y	Y	Y	Y	U	Y	Y	Y	Y	Y	Y	Y	Y	92,3	12	0	1

*Legend*: *Y* Yes, *N* No, *U* Unclear

Table 2 summarises the characteristics of the studies.

**Table 2:** Summary table of studies included in the systematic review of the literature

Authors, year, reference	Country of study	Title, participants	Objective	Methodology	Interventions	Description of the main results/conclusions
Bendtsen et al. 2020 [24]	Sweden	**Title**—A Mobile Health Intervention for Mental Health Promotion Among University Students: Randomised Controlled Trial **Participants** – *N* = 654. University students, who could read and understand Swedish; and had access to a mobile phone (intervention group: *N* = 348; control group: *N* = 306; mean age: 25 years)	To estimate the effect of a fully automated mHealth intervention on positive mental health, and anxiety and depression symptomatology among Swedish university students using a randomised controlled trial design	Randomised control trial. A 2-arm, single-blind (researcher), parallel groups. The programme lasted 10 weeks. A new topic was introduced each week	Intervention group exposed to a mHealth positive psychology programme Control group exposed to relevant online mental health information The intervention was a fully automated mHealth positive psychology multi-component programme. The programme included wellbeing information, validated self-help exercises, short tips, self-monitoring and personalised feedback. Text messages were sent to users throughout the programme, with an average of one text message per day, containing text and links to interactive exercises and further reading	Using an automated mobile phone format improved positive mental health. The mHealth intervention was estimated to be superior to usual care in increasing positive mental health among university students The intervention also had a protective effect on depressive and anxiety symptoms. These findings demonstrate the feasibility of using an automated mobile phone format to improve positive mental health, which holds promise for the use of mHealth solutions in public mental health promotion
Hall et al. 2018 [25]	China	**Title –** An evaluation of a low intensity mHealth enhanced mindfulness intervention for Chinese university students: A randomised controlled trial **Participants—** *N* = 101. University students (intervention group: *N* = 76; control group: *N* = 25; mean age: 22.3 years)	To evaluate a low-cost scalable mindfulness intervention programme to improve psychological health and sleep quality in Chinese university students	Randomised controlled trial. Participants were randomised to 4 groups: Group 1: control group (*N* = 25), Group 2: (*N* = 27), Group 3: (*N* = 24), and Group 4: (*N* = 25)	Low-cost, scalable mindfulness intervention programme to improve mental health and sleep quality Intervention groups consisted of: group 2—mindfulness only; group 3—mindfulness with plain-text reminders; group 4—mindfulness with enhanced text reminders featuring animal memes The mindfulness intervention included two in-person guided sessions and seven weeks of weekly self-guided practice	The findings suggest that low-intensity mindfulness interventions may be a useful programme in university settings
Wong et al. 2021 [26]	China	**Title –** An Interactive Web-Based Sexual Health Literacy Program for Safe Sex Practice for Female Chinese University Students: Multicenter Randomised Controlled Trial **Participants—** *N* = 746. Female Chinese university students at 5 dormitories in Hong Kong (intervention group: *N* = 362; control group: *N* = 384; mean age: 21.5 years)	Describes the development and systematic evaluation of a web-based sexual health literacy intervention called “Smart Girlfriend” for female Chinese university students	Multicentre randomised controlled trial. Participants were randomised to 2 groups	The development and systematic evaluation of a web-based sexual health literacy intervention called "Smart Girlfriend" Intervention group—received an interactive web-based sexual health literacy intervention Control group—received a single web page of online information about condom use The intervention content was based on the Health Belief Model and the Continuum of Conflict and Control theory	An interactive web-based sexual health literacy programme did not significantly increase condom use consistency compared to a single webpage of condom use information, but it did temporarily improve knowledge, attitudes, norms and self-efficacy regarding condom use
Saleh et al. 2021 [27]	France	**Title –** Can we learn to manage stress? A randomised controlled trial carried out on university students **Participants—** *N* = 128. University students (intervention group: *N* = 64; control group: *N* = 64; mean age: 21.54 years)	Measure the efficacy of an online stress management program for university students based on several mental health variables: self-esteem, perceived stress and its two subfactors (perceived helplessness and perceived self-efficacy), psychological distress and its four subfactors (somatic symptoms, anxiety/insomnia, social dysfunction, severe depression) and satisfaction with studies	Randomised controlled trial. Subjects were randomly assigned to one of two groups	Online stress management programme called “I’m managing my stress” Intervention group: online programme consisted of four sessions of 20 min each, including psychoeducation, practical exercises and one to two weekly activities that the participant is asked to complete (task prescription as in cognitive behavioural techniques). The aim was for the participants to learn simple techniques to help them cope better with stressful situations Control group: did not follow the programme	This type of internet-based programme has the potential to reach many students due to its relatively short format and accessibility. It has already shown improvements in levels of perceived stress, psychological distress and satisfaction with study The option of online interventions could appeal specifically to students who do not seek professional help
Apolinário-Hagen et al. 2021 [28]	Germany	**Title** – Exploring the influence of testimonial sources on attitudes towards e-mental health interventions among university students: Four-group randomised controlled trial **Participants –** *N* = 451. University students (intervention group: *N* = 339; control group: *N* = 112, mean age: 32.6 years)	To explore the extent to which different ways of targeting students with information affect their attitudes towards eMHS for stress prevention and treatment, and to identify potential determinants of attitude change	Randomised controlled trial	Transmission of only general information on the eMHS to the participants Control group: arm 1—participants only received general information about electronic mental health services Intervention groups: arm 2: transmission of general information to participants about SSME's and non-specific testimonies; arm 3: participants received general information about eMHSs and work-related testimonials; arm 4: transmission of general information to participants about SSME's and testimonies directed at student workers	Overall, this study found no significant effect of information on attitudes and limited evidence for the benefits of tailored narrative messages
Heeren et al. 2017 [29]	United States of America	**Title –** Health-Promotion Intervention Increases Self-Reported Physical Activity in Sub-Saharan African University Students: A Randomised Controlled Pilot Study **Participants –** *N* = 176. University students (intervention group: *N* = 85; control group: *N* = 91, mean age: 20.84 years)	To evaluate the efficacy of a health-promotion intervention in increasing self-reported physical activity among university students in Sub-Saharan Africa	Randomised control trial	Each intervention consisted of 8, 45-min modules, with 2 modules implemented during each of 4 weekly sessions. Each intervention was highly structured and involved interactive exercises, games, brainstorming, role-playing, videos, and group discussions implemented in mixed-gender groups of 7 to 11 participants (mean group size = 8.8) led by co-facilitator pairs using standardised intervention manuals The health-promotion intervention was designed to increase knowledge, attitudes, self-efficacy, and skills to prevent NCDs by increasing physical activities, choosing healthy diets, and limiting alcohol use Control group: participants exposed to an intervention to reduce the risk of HIV, with the reduction of unprotected sex and multiple sexual partners, with 8 modules	The findings suggest that theory-based, contextually appropriate interventions may increase physical activity among university students in Sub-Saharan Africa Participants in the health promotion intervention were more likely to meet physical activity guidelines Health promotion participants reported a greater number of days of vigorous-intensity and moderate-intensity aerobic activity, but not of muscle-strengthening activity. The intervention reduced self-reported servings of fried foods
Kim et al. 2018 [30]	United States of America	**Title –** Promoting physical activity using a wearable activity tracker in college students: A cluster randomised controlled trial **Participants –** *N* = 187. College students (intervention group: *N* = 101; control group: *N* = 86, mean age: 20.09 years)	To investigate the effects of using a wearable activity tracker in a credit-based physical activity instruction programme (PAIP) to promote physical activity (PA) in college students	Randomised controlled trial in which students from fourteen courses of the Physical Activity Instruction Programme (PAIP) at a large public university	Participants in the intervention groups were provided with a small, lightweight activity tracker, the Misfit Flash, which can be worn with a clasp or wristband. A handout was provided with step-by-step instructions on how to use the activity tracker with the Misfit app on smartphone The detailed features of the Misfit app include: activity tracking (total steps, calories burned, distance run and/or walked, and activity points calculated from these three measures), personalised goal setting, standardised behavioural feedback, and social community support, which are the essential components facilitating self-directed behavioural change. Control group participants received no additional instruction beyond the scheduled classroom activities based on the standardised PAIP core curriculum	Taken together, the present study found null effects of using the wearable activity tracker to promote physical activity in college students, suggesting that primary intervention using the wearable activity tracker as a behaviour change strategy may not be effective in increasing physical activity in this setting
Rosenberg & Hamiel 2018 [31]	Israel	**Title**—Reducing Test Anxiety and Related Symptoms Using a Biofeedback Respiratory Practice Device: A Randomised Control Trial **Participants –** *N* = 22. University students (intervention group: *N* = 17; control group: *N* = 5, mean age: 25.10 years)	Investigate simple self-help interventions to reduce test anxiety	Randomised controlled trial. It investigated a simple behavioural intervention—the use of breathing tools—as an exclusive therapy for test anxiety. Specifically, the effectiveness of a biofeedback breathing training device was investigated	Behavioural and psychoeducational interventions. Intervention groups: 2 intervention groups were created: the biofeedback device group and the self-directed breathing exercise group Participants completed the Test Anxiety Inventory (TAI) and were given the CalmiGo® device to use for two weeks, after which they were told they would take part in a simulation of an intelligence test. Control group: participants who received the same psychoeducation and general instructions as the other 2 groups	The findings support the notion that the use of biofeedback respiratory devices can reduce students' test anxiety symptoms
Viskovich & Pakenham 2019 [32]	Australia	**Title** – Randomised controlled trial of a web‐based Acceptance and Commitment Therapy (ACT) program to promote mental health in university students **Participants –** *N* = 437. University students (intervention group: *N* = 249; control group: *N* = 188, mean age: 26.85 years)	To evaluate the effectiveness of a web‐based ACT mental health promotion intervention, called YOLO (You Only Live Once)	Randomised controlled trial, in which participants were randomised to the intervention or to the waiting list	Intervention group: exposure to the web-based mental health promotion programme called YOLO for 4 weeks. The web-based intervention for college students (animated presentations, video clips, audio files, and written exercises) consisted of four 30- to 45-min sessions of 4 modules, each targeting one or two of the six core processes of Acceptance and Commitment Therapy (ACT). There was no face-to-face contact with the programme facilitators. The topics of the modules were: Module 1—Values and Commitment, Module 2—Cognitive Defusion, Module 3—Acceptance, and Module 4—Mindfulness and 'I as Context'. The exercises in the programme lasted from 5 to 15 min Control group: participants on the waiting list (completed the assessment instruments at the same time as those in the intervention group). They did not receive any intervention	The results support a web-based ACT intervention to promote mental health in university students Analyses showed significant improvements from pre- to post-intervention compared to wait-list control on all primary outcomes and ACT processes. All intervention gains were maintained at follow-up. Improvements in all primary outcomes were mediated by three or more ACT processes in both samples. Intervention effects were consistent in both sample groups

### Types of intervention studies

Of the included studies, five developed programmes [24–27, 32] and 4 evaluated the effectiveness of interventions in improving outcomes [28–31].

The studies included interventions aimed at higher education students based on the transmission of information on a topic related to health and with the aim of developing competences in the participants to understand the information and adopt healthy behaviours that protect health. The forms of information transmission aimed at simplifying and improving information materials (use of simple language, simplified texts, illustrations, videos, animated presentations, video clips, audio files, pictograms, icons, memes, meaningful formats, personalised information, and the development of materials that are easy to read and use). The programs and interventions designed included prescriptive exercises or written exercises, self-monitoring, and monitoring by a health professional The interventions were carried out individually or in groups [24–32].

The duration of the various interventions was between four [32] and sixteen weeks [32]. The health literacy interventions contents were based on the Health Belief Model [26], theories and empirical evidence from the positive psychological research field, cognitive behavioural techniques [26], transactional theory of stress [26], theory of planned behaviour [27, 28], technology acceptance model [27], acceptance and commitment therapy [32], continuum of conflict and control theory [26], behaviour change theories [29], and social cognitive theory [29, 29].

Health literacy interventions for higher education students were developed in various formats including websites, leaflets, smartphone apps, written messages, and person delivered [24–32]. Interventions provided education, information (about online services or health topics), empowerment (stress management, physical activity, sexual disease prevention, and effective sleep), education (about healthy eating or condom use), persuasion (about healthy behaviours and lifestyles), and anxiety relief (managing symptoms of anxiety, stress, and depression). Outcomes measured were increase in knowledge, increase in confidence/patient activation, and change in behaviour [24–32].

### Themes of the health literacy interventions

The analytic themes identified in the programmes or interventions were mental health [24, 25, 27, 32], physical activity [29, 30], sexual health literacy [26], use of electronic mental health services [28], sleep quality [25], and test anxiety [31]. Mental health was the only theme, with the following subthemes: (i) mental health promotion [24, 25, 27, 32], and (ii) use of electronic mental health services [28]. For the theme of physical activity, we identified the sub-theme of promotion of physical activity [29, 30]. For the theme of sexual health, we identified the sub-theme of prevention of sexually transmitted diseases [26].

When we relate these themes and subthemes with the theoretical model of Sørensen et al. [1], a reference for the present analysis, for the health promotion dimension, we identified four studies on mental health promotion [24, 25, 27, 32] and two on physical activity promotion [29, 30]. Regarding disease prevention, we identified one study on reducing test anxiety and related symptoms [31] and one study on preventing sexually transmitted diseases [26]. As for the topic of using services, we identified one study on the use of electronic mental health services [28].

### Effectiveness of the health literacy interventions on health gains

#### Effectiveness in health promotion

In assessing the effect of an intervention developed using a mobile application on positive mental health and anxiety and depression symptoms, it was found that exposing students to an automated mobile application had a positive mental health effect, and they had lower symptoms of depression and anxiety compared to participants in the control group. They also observed a high level of social, emotional, and psychological well being [24]. A study for evaluating the effectiveness of a mental health promotion intervention through acceptance and commitment therapy, found a decrease in the level of stress, improvement in well-being, self-compassion, life satisfaction, and academic performance. It also verified positive results in the processes of acceptance and commitment: acceptance, cognitive fusion, educational values, valued life, and awareness of the present moment [32].

Evaluating a low-intensity mHealth intervention optimised with mindfulness, with a mobile application, showed that participants exposed to low-intensity interventions significantly reduced the symptomatology of depression and anxiety and improved subjective sleep quality, sleep latency, and usual sleep efficiency [25]. Regarding stress control, a study evaluated a self-help programme developed over the internet for stress management and found that there was an improvement in the level of perceived stress, psychological distress, and increased satisfaction with studies. They concluded that some students do not seek these services in person but participate online [27].

In a study evaluating the effectiveness of a health promotion intervention to increase self-reported physical activity in university students in sub-Saharan Africa, interactive exercises, games, brainstorming, role-playing, videos, and group discussions, led by pairs of co-facilitators, using intervention manuals, found that participating students increased levels of physical activity, increased consumption of fruits and vegetables and even reduced consumption of fried foods [29]. Aiming to promote physical activity in university students, it was investigated the effect of a wearable physical activity tracker integrated into a physical activity instruction programme on physical activity. The results demonstrated a null effect between the use of the tracker and an increase in physical activity [30].

#### Effectiveness in disease prevention

Regarding disease prevention, we included two studies in this analysis point: one on the prevention of anxiety and related symptoms [31] and the other on the prevention of sexually transmitted diseases [26].

The effect of using a breathing biofeedback device to prevent "test anxiety" by assessing anxiety, depression, and stress in university students found that use of the device reduced anxiety, depression, and stress [31]. A study aimed to describe the development and systematic evaluation of a web-based sexual health literacy intervention called “Smart Girlfriend” for female Chinese university students, concluded that the programme did not significantly increased the consistency of condom use compared to a single webpage of condom use information; however, it did temporarily improved knowledge, attitudes, norms, and self-efficacy regarding condom use [26].

#### Effectiveness in health care services application

In the health service dimension, we identified only one study with outcome associated with health service application. The study aimed to explore how far different ways of targeting information to students affect their attitudes towards electronic mental health services for stress prevention and therapy, and to identify potential determinants of attitude change, indicated no meaningful impact of information on attitudes and limited evidence for benefits of tailored narrative messages [28].

### Health gains sensitive to health literacy interventions

In the studies, we determined the health gains (positive changes in health outcomes) of higher education students after health literacy interventions.

Health literacy intervention characteristics and resulting health gains are shown in Table 3.

**Table 3:** Characteristics of health literacy interventions and resulting health gains

**Authors, year, reference**	**Health literacy level according to the Nutbeam model **[5]	**Health literacy dimension**	**Intervention type**	**Individual benefits (knowledge, motivation, and skills)**	**Health gains**
Bendtsen et al. 2020 [24]	Functional, Interactive	Health promotion	Web based Tailored information	Knowledge about positive mental health Ability to act independently, increasing motivation and confidence to act on mental health advice	↑ levels of positive mental health ↑ emotional, social and psychological wellbeing ↓ levels of stress and anxiety
Hall et al. 2018 [25]	Functional, Interactive	Health promotion	Tailored information	Knowledge of mental health and sleep quality Motivation and confidence to act on advice received can increase ability to act independently	↑ subjective sleep quality, sleep latency and habitual sleep efficiency ↓ depression, anxiety and stress
Wong et al. 2021 [26]	Functional, Interactive	Disease prevention	Web based	Knowledge about sexual health information Self-confidence to act on advice received can increase one's capacity to act independently	↔ Consistency of condom use ↑ knowledge, attitudes, norms, and self-efficacy for condom use
Saleh et al. 2021 [27]	Functional, Interactive	Health promotion	Web based	knowledge of stress management Confidence to act on advice received can increase ability to act independently (cope better with stressful situations)	↑ self-esteem, perceived stress, satisfaction with studies, and in the somatic symptoms, anxiety and insomnia and severe depression
Apolinário-Hagen et al. 2021 [28]	Interactive	Healthcare	Only information transmission	Confidence in acting on received advice can increase the ability to use electronic mental health services independently	↑ influences of source credibility and perceived similarity on attitudes towards preventive eMHS ∅ information on changing attitudes towards eMHS for coping with stress
Heeren et al. 2017 [29]	Functional	Health promotion Disease prevention	Multi-component programme	Knowledge of the importance of health promotion and disease prevention	↑ knowledge of physical activity guidelines ↑ number of days of vigorous and moderate-intensity aerobic activity, but not strength-building activity ↓ self-reported servings of fried foods
Kim et al. 2018 [30]	Interactive	Health promotion	Information on numerical concepts	Ability to act independently in promoting physical activity	∅—physical activity at moderate or vigorous intensity
Rosenberg & Hamiel 2018 [31]	Interactive	Health promotion	Group-based	Ability to act independently to reduce test anxiety Confidence to act on advice received	↑ psychological wellbeing ↓ test anxiety symptoms ↓ symptoms of depression and anxiety
Viskovich & Pakenham 2019 [32]	Interactive	Health promotion	Web based	Ability to act independently to promote mental health Motivation to promote mental health	↑ increased well-being, self-compassion and academic performance ↓ symptoms of depression, anxiety and stress

*Legend*: ↑—Increased; ↓—Decreased; ↔—Not significant; ∅—null effect

The health gains sensitive to health literacy interventions were observed at mental health [24, 25, 27, 31, 32], wellbeing [24, 31, 32], sleep quality, sleep latency and habitual sleep efficiency [25], attitudes for preventative electronic mental health services [28], knowledge, attitudes, and self-efficacy for condom use [26], physical activity [29], and healthy eating [29].

The health literacy intervention typology that had the greatest impact on health gains was electronic interventions that involved interaction (between peers or promoters) with diverse methodology.

Providing information on condom use or the use of a physical activity tracker did not prove effective for behaviour change [26]. Conversely, electronic health literacy interventions had a positive effect on mental health, depression, anxiety and stress symptoms, feelings of stress, psychological distress, and satisfaction with studies, and reduced symptoms of test anxiety in students [24, 25, 27, 29, 31].

According with the main conclusions of the studies there was evidence:

1. A mobile health intervention for mental health promotion was estimated to be superior to usual care in increasing positive mental health [24]
2. A low intensity mHealth enhanced mindfulness intervention might be a useful intervention programme [25]
3. An Interactive Web-Based Sexual Health Literacy Program for Safe Sex Practice for Female Chinese University Students did not significantly increase the consistency of condom use compared to a single webpage of condom use information; however, it did temporarily improve knowledge, attitudes, norms, and self-efficacy regarding condom use [26]
4. An Internet-based program to learn to manage stress has the ability to reach a large number of students due to its rather short format and accessibility. It has already shown improvements in terms of the levels of perceived stress, psychological distress, and satisfaction with studies [27]
5. Testimonial source on attitudes towards e-mental health interventions indicated no meaningful impact of information on attitudes and limited evidence for benefits of tailored narrative messages [28]
6. Health-Promotion Intervention may increase Physical Activity [29]
7. Promoting physical activity using a wearable activity tracker have a null effect in physical activity [30]
8. Using biofeedback respiratory devices may reduce students’ Test Anxiety symptoms [31]
9. A web‐based Acceptance and Commitment Therapy (ACT) program promotes mental health for university students [32].

## Discussion

This systematic review assessed the evidence on the effectiveness of health literacy interventions in higher education. We conducted a qualitative analysis with thematic and narrative synthesis because there were not multiple studies with similar outcome measures or similar interventions to conduct a quantitative data analysis (meta-analysis or statistical pooling). Most studies focused on assessing the efficacy of a given intervention on lifestyle and its effects, particularly on mental health, physical activity, mental health services, sleep quality, healthy eating, and quality of life. To our knowledge, there are no other systematic reviews on this topic with which to compare the results. There are systematic reviews that have been conducted with adults, but not with university students.

Unhealthy behaviours are risk factors for noncommunicable diseases such as cardiovascular diseases, cancer, diabetes, and respiratory diseases [34]. Many risk factors for these diseases such as obesity, tobacco consumption, inadequate diet, stress, and physical inactivity are modifiable [34]. Interventions to promote healthy lifestyles can be an opportunity to change behaviour [34]. With regard to students in higher education health literacy activities should be targeted at students of all courses, and university resources should be used to provide health literacy courses for students, in line with university course provision [14].

There were two intervention types: with peer or promoter interaction in six studies [24, 25, 27, 29, 31, 32] and without interaction with simple information transfer in three studies [26, 28, 30]. Educational and motivational interventions were used in all studies to modify or promote health outcomes [24–32]. Cognitive and behavioural interventions using digital technology that allowed appropriate interaction (between peers or promoters) and feedback were most effective. In one study [30], there was no evidence to support the use of a wearable activity tracker to promote physical activity. The interventions in this study were the use of a small, lightweight activity tracker (“Misfit Flash”), which can be worn with a clasp or watch band, in combination with a Misfit smartphone app and planned class activities based on the Physical Activity Instruction Program standardised core curriculum.

Regarding the use of technology in interventions, all studies [24–32] used electronic devices such as computer software, web page, electronic texting message, respiratory device, monitoring software, or smartphone apps. Interventions using a single method that emphasised the transmission of information by promoters did not show changes in outcomes despite the use of the Internet and devices [26, 28, 30]. Interventions using different methods, with exercises, with peer monitoring, or with feedback from promoters showed positive results in outcomes [24, 25, 27, 29, 31, 32]. An effective public health strategy includes the use of wearable devices as a psychological intervention for healthy lifestyles (more effective for lifestyle changes) [35]. One study with 485 Portuguese university students found that the most popular way for students to access information was through the internet, which was also associated with the lowest levels of health literacy [36]*.* One of the reasons for this is the quality of the information available on the Internet, which is often inaccurate and difficult to understand [14].

A positive aspect of this review is that all studies were randomised controlled trials with a comparison group, which is useful for comparing the effectiveness of interventions. Study design, measurement tools, and health outcomes were not similar. We found the same instruments to measure primary and secondary health outcomes, for example Depression, Anxiety, and Stress Scale (DASS) [25, 31, 32] and Perceived Stress Scale (PSS) [27, 28], but the type of interventions was different. It was difficult to relate the type of intervention to health outcomes and health gains.

The quality of the studies was good, but the heterogeneity of the subjects and the design of the interventions limit the generalizability of the results [24–32]. The studies were designed to improve outcomes such as knowledge, motivation, and confidence, which contribute to better health outcomes and health gains. Interventions targeted interactive skills and others with interactive and critical thinking skills, rather than just teaching information to improve knowledge. Using the internet and gamification approaches are options for delivering interesting health literacy interventions to higher education students, and social networks can also provide an easy way to reach and connect students to peer-to-peer programmes [14]. The design of health literacy interventions should take into account the different needs and characteristics of subgroups of students in order to increase their effectiveness [14].

Regarding health gains, the interventions in the studies selected for this review resulted in several health gains. A study aimed at improving users' positive mental health with a fully automated mHealth multicomponent programme, based on theories and empirical findings from the field of positive psychology research and developed over a 10-week period, was found to be superior to usual care in improving the positive mental health of university students [24]. The intervention was also found to have a protective effect on depressive and anxiety symptoms [24]. A study evaluating a low-cost, scalable mindfulness intervention programme to improve mental health and sleep quality among Chinese university students over a seven-week period may be a useful intervention programme in the university setting [25].

An interactive web-based sexual health literacy programme for safer sex practices did not significantly increase condom use consistency compared to a single condom information webpage, but it did temporarily improve knowledge, attitudes, norms and self-efficacy regarding condom use [26]. As the internet is the preferred way to obtain health information even if it does not lead to better health literacy or eHealth literacy, work is needed to promote the quality of the information and the ability of students to evaluate it [36].

An online stress management programme for university students showed improvements in perceived stress, psychological distress, and satisfaction with studies [27]. Online interventions could specifically target students who do not seek professional help [26]. An investigation of the influence of different types of specific information provided to students on their attitudes towards electronic mental health services (eMHS) for the prevention and treatment of stress, and the identification of potential determinants of attitude change, found no significant effect of information on attitudes and limited evidence of the benefits of tailored narrative messages [27].

To assess the effectiveness of a health promotion intervention in increasing self-reported physical activity among university students in sub-Saharan Africa, a study suggests that theory-based and contextually appropriate interventions can increase physical activity and reduce self-reported servings of fried foods [28]. Students will be able to understand the risks associated with unhealthy foods and how to avoid eating them in everyday life [37]. Using a wearable activity tracker in a credit-based physical activity instructional program (PAIP) to promote physical activity (PA) among college students found null effects of using the wearable activity tracker to promote PA [30].

A study investigating simple self-help measures to reduce test anxiety found that only participants who used a biofeedback device experienced a significant reduction in test anxiety symptoms, as well as a reduction in symptoms of depression and anxiety and an increase in psychological well-being, a subscale of the Quality of Life Questionnaire [31]. The use of biofeedback respiratory devices can reduce symptoms of test anxiety in university students [31].

In the area of mental health promotion, the results of a study evaluating the effectiveness of a web-based Acceptance and Commitment Therapy (ACT) programme for mental health promotion found the programme to be important [32].

There were strengths in this review: (i) the evidence base was generally recent (about half of the included studies had been published since 2020) [24, 26, 28, 31], (ii) all studies were randomised controlled trials (a group for comparison was essential to the aim of this revision) [24–32], (iii) there were diversity themes, and (iv) the methodology quality of the studies. This review also had limitations: (i) we desired more studies with comparison groups for generalisation of the results, (ii) the details of interventions like the theory or model were not identified in all interventions, (iii) outcome measurements were heterogeneous, we suggest an outcome like health literacy level associated to health outcomes, and (iv) weren’t possible a statistical pool construction for meta-analysis.

The co-design of health literacy interventions it was not explicit in some studies. We found interventions that used psychological theories, but we did not have enough examples or enough information about the development processes to determine if taking these approaches was more likely to result in effective interventions. The results of this review cannot readily be generalised, and its interpretation should only be applied in the study context. Health literacy is a developing field with very few interventions using clear theoretical frameworks. Closer links between health literacy and behaviour change theories and frameworks could result in higher quality and more effective interventions [38].

### Implications for research

In our synthesis we used quantitative studies with a comparison group. In future syntheses, we think it would be beneficial to include studies of a different type. It would also be interesting to explore interventions that translate into health gains in contrasting populations (different cultures, socioeconomically disadvantaged, and rural and urban populations) [39].

### Implications for practice

Knowing the characteristics of health literacy interventions that have an impact on health outcomes among higher education students allows for better planning of interventions at the design level. With regard to the implementation of interventions, we found that the simple delivery of information is not significant for behaviour change. We suggest that the development of interventions should combine educational methods that are useful for understanding and using health content. Providing credible health content should be a concern so that students have access to this information but are also able to understand and use it. In our review, we found that health literacy interventions should not be limited to providing credible information but should also assess its understanding and support its use.

### Implications for policy

The results of this systematic review are also relevant for policy makers. We found that health interventions delivered in an academic setting had an impact on health outcomes. Our findings highlight the potential benefits of health literacy interventions developed in academic settings for higher education students. By promoting and supporting health literacy interventions for their students, higher education institutions are contributing to a future of more empowered adults by preparing them for health needs and increasing their capacity to promote their health, prevent illness and access health care.

There is growing evidence that health literacy is about enabling and empowering people to protect and care for their health, and creating an enabling and supportive environment for evidence-based health decision-making. In this context, health literacy is widely recognised as a key component in addressing current complex public health issues [40].

## Conclusions

This systematic review contributes to increasing knowledge on the efficacy of the health literacy interventions on health outcomes, suggesting interventions for future investigations. The results of this systematic review are a reference for health literacy interventions at higher education students, in the domains of health promotion, disease prevention or health care.

Health literacy interventions that combined interaction, exercises, goals definition, technology use, monitoring and mentoring were more effective to health outcomes than a simple information transmission. Future studies should explore the effectiveness of the interventions to health literacy level. For future we suggest similar literature reviews like this one. Health literacy interventions developed in academic settings delivered to higher education students influence positive health outcomes. Mental health is the theme more studied, and the health gain was depression, anxiety, and stress management. Health literacy interventions improved mental health; decreased depression, anxiety and stress symptoms; increased well-being (emotional, social and psychological); increased physical activity; improved sleep quality; improved knowledge, attitudes, norms and self-efficacy regarding condom use; and decreased consumption of fried foods.

Health literacy interventions in higher education students can significantly improve health outcomes and protect them from the negative effects of health threats. Interventions designed with different strategies are more effective. For higher education students’ attendance to be a successful experience, continuity of health literacy interventions developed in the academic setting is necessary. Understanding the specifics of health literacy interventions and the health benefits they provide to higher education students is important to the health planning process and to the public health mission.

## Data Availability

All data generated or analysed during this study are included in this published article. This article is a secondary study – a systematic review of primary studies. The primary studies included in the systematic review are described in the references  [24–32]. Other data generated in this study and data collection templates are available from the corresponding author upon reasonable request.

## Abbreviations

HL: Health Literacy
HP: Health Promotion
DP: Disease Prevention
HC: Health care
DOI: Digital Object Identifier
Y: Yes
N: No
U: Unclear
HP: Health Promotion
DP: Disease Prevention
HC: Health Care
